# Diseases of the Rectum and Anus

**Published:** 1893-03

**Authors:** 


*Diseases of the Rectum and Anus.* By ALFRED COOPER, F.R.C.S., and F. SWINFORD EDWARDS, F.R.C.S. Second edition. Pp. xix., 324. London: J. & A. CHURCHILL. 1892.

The surgery of the rectum has undergone considerable development during late years, and books that were written some years ago have to undergo much alteration to bring them up to date. The present edition has been carefully revised by the authors, and includes the most approved modern method of treatment.

A very good and full account of the various ways of excising the rectum is given, and approval is expressed of the preliminary operation of inguinal colotomy in many cases of extensive disease.

We are somewhat surprised at the very scanty notice of the operation for fistula-in-ano by excision and immediate suture, the authors not having treated any cases by this method. We notice also that they still believe in the ligature as the best method of treating hemorrhoids. In both of these particulars they illustrate the intense conservatism of the London schools. But upon the whole the work is distinctly good. It is written in a pleasant style, and is illustrated by several good photographic plates and numerous engravings.

